# Supplementary material for: A cluster feasibility trial to explore the uptake and use of e-cigarettes versus usual care offered to smokers attending homeless centres in Great Britain
Source: PLoS One. 2020 Oct 23;15(10):e0240968. doi: 10.1371/journal.pone.0240968 (PMC7584191; doi:10.1371/journal.pone.0240968)
Supplement: S1 Table — (DOCX) [file pone.0240968.s002.docx]

**S1 Table**

|  | **EC** | | | **UC** | | | **Total** |
| --- | --- | --- | --- | --- | --- | --- | --- |
|  | Centre 1 Midlands  (day centre) | Centre 4  London  (residential) | **EC total** | Centre 2  Edinburgh  (day centre) | Centre 3  London  (residential) | **UC total** |  |
| Eligible participants invited: N | 75 | 15 | 90 | 45 | 18 | 63 | 153 |
| Consented: N (% of those invited) | 39 (52%) | 9 (60%) | 48 (53.3%) | 23 (51.1%) | 9 (50%) | 32 (50.8%) | 80 (52.3%) |
| 4 week retention: N (% of those consented) | 33 (84.6%) | 6 (66.7%) | 39  (81.3%) | 14 (60.9%) | 7 (77.8%) | 21 (65.6%) | 60 (75%) |
| 12 week retention: N (% of those consented) | 30 (76.9%) | 4 (44.4%) | 34 (70.8%) | 11 (47.8%) | 7 (77.8%) | 18 (56.3%) | 52  (65%) |
| 24 week retention: N (% of those consented) | 29 (74.4%) | 6 (66.7%) | 35 (72.9%) | 6 (26.1%) | 6 (66.7%) | 12 (37.5%) | 47  (58.8%) |
