# Supplementary material for: A cluster feasibility trial to explore the uptake and use of e-cigarettes versus usual care offered to smokers attending homeless centres in Great Britain
Source: PLoS One. 2020 Oct 23;15(10):e0240968. doi: 10.1371/journal.pone.0240968 (PMC7584191; doi:10.1371/journal.pone.0240968)
Supplement: S2 Table — (DOCX) [file pone.0240968.s003.docx]

**S2 Table**

|  | **Baseline** | **4-week FU** | **12-week FU** | **24-week FU** |
| --- | --- | --- | --- | --- |
| **N = 39***  **^+^Sharing cigarettes:**  **N (%)** |  |  |  |  |
| Not at all | 20 (51.3) | 21 (53.8) | 22 (56.4) | 18 (46.2) |
| Occasionally | 6 (15.4) | 11 (28.2) | 13 (33.3) | 10 (25.6) |
| Regularly | 2 (5.1) | 2 (5.1) | 3 (7.7) | 5 (12.8) |
| Daily | 11 (28.2) | 5 (12.8) | 1 (2.6) | 6 (15.4) |
| **Smoked discarded cigarettes: N (%)** |  |  |  |  |
| Not at all | 24 (61.5) | 31 (79.5) | 34 (87.2) | 30 (76.9) |
| Occasionally | 11 (28.2) | 1 (2.6) | 3 (7.7) | 4 (10.3) |
| Regularly | 0 | 3 (7.7) | 1 (2.6) | 2 (5.1) |
| aily | 4 (10.3) | 3 (7.7) | 1 (2.6) | 2 (5.1) |
| **Asked a stranger for a cigarette: N (%)** |  |  |  |  |
| Not at all | 26 (66.7) | 33 (84.6) | 31 (79.5) | 35 (89.7) |
| Occasionally | 9 (23.1) | 4 (10.3) | 7 (17.9) | 2 (5.1) |
| Regularly | 2 (5.1) | 0 | 1 (2.6) | 1 (2.6) |
| Daily | 2 (5.1) | 1 (2.6) | 0 | 0 |

*Sample includes only those who came back for all follow-up sessions (n=39). ^+^Sharing cigarettes is referred to here as smoking from the same cigarette (not sharing an unsmoked cigarette from a packet).
