# Supplementary material for: A cluster feasibility trial to explore the uptake and use of e-cigarettes versus usual care offered to smokers attending homeless centres in Great Britain
Source: PLoS One. 2020 Oct 23;15(10):e0240968. doi: 10.1371/journal.pone.0240968 (PMC7584191; doi:10.1371/journal.pone.0240968)
Supplement: S3 Table — (DOCX) [file pone.0240968.s004.docx]

**S3 Table**

|  | UC (n=32) | EC (n=48) |
| --- | --- | --- |
| SSS session attendance | 4 weeks: 1 attended 2, 3 attended 1 12 weeks: 1 attended 1, 1 attended 2 24 weeks: 1 attended 1, 1 attended 2 | No one attended any at 4, 12 and 24 weeks |
| Other medication supplied by SSS | 4 weeks: 3 (inhaler, patches, lozenges) 12 weeks: 2 (patches, lozenges, mints) 24 weeks: 1 (gums, patches) | No one used any at 4, 12 and 24 weeks |
| Smoking cessation from primary care professionals | Baseline: 1 sought GP once, 1 sought practice nurse once | Baseline: 3 sought GP 1-2 times, 2 sought practice nurse once, 1 sought pharmacist once |
|  | 4 weeks: 2 sought GP once, 1 sought practice nurse once, 3 sought pharmacist 1-3 times | 4 weeks: none |
|  | 12 weeks: none | 12 weeks: 1 sought GP once, 1 sought practice nurse once, 1 sought pharmacist once |
|  | 24 weeks: none | 24 weeks: 1 sought GP once |
| NRT on prescription | Baseline: 1 (patches) 4 weeks: 2 (patches, lozenges, inhaler) 12 weeks: 2 (patches, lozenges) 24 weeks: 1 (patches, gums) | Baseline: none 4 weeks: none 12 weeks: none 24 weeks: 1 (nasal spray) |

UC: baseline n=32, 4 weeks n=21, 12 weeks n=18, 24 weeks n=12; EC: baseline n=48, 4 weeks n=39, 12 weeks n=34, 24 weeks n=35
