# Supplementary material for: A cluster feasibility trial to explore the uptake and use of e-cigarettes versus usual care offered to smokers attending homeless centres in Great Britain
Source: PLoS One. 2020 Oct 23;15(10):e0240968. doi: 10.1371/journal.pone.0240968 (PMC7584191; doi:10.1371/journal.pone.0240968)
Supplement: S4 Table — (DOCX) [file pone.0240968.s005.docx]

**S4 Table**

|  | **Tobacco 18 mg/mL** | **Tobacco 12 mg/mL** | **Menthol 18 mg/mL** | **Menthol 12 mg/mL** | **Fruit**  **18 mg/mL** | **Fruit**  **12mg/mL** |
| --- | --- | --- | --- | --- | --- | --- |
| Baseline | 34 | 35 | 22 | 25 | 59 | 60 |
| Week 1 | 10 | 14 | 16 | 27 | 29 | 51 |
| Week 2 | 8 | 13 | 17 | 17 | 17 | 42 |
| Week 3 | 2 | 17 | 17 | 14 | 16 | 44 |
| Total | 54 | 79 | 72 | 83 | 121 | 197 |

mg/mL = nicotine concentration in milligrams of nicotine per mL of e-liquid
