# Supplementary material for: A cluster feasibility trial to explore the uptake and use of e-cigarettes versus usual care offered to smokers attending homeless centres in Great Britain
Source: PLoS One. 2020 Oct 23;15(10):e0240968. doi: 10.1371/journal.pone.0240968 (PMC7584191; doi:10.1371/journal.pone.0240968)
Supplement: S1 Protocol — (DOCX) [file pone.0240968.s007.docx]

**Exploring the uptake and use of electronic cigarettes provided to smokers accessing homeless centres: a feasibility study**

Sharon Cox^1^, Linda Bauld^2^, Allison Ford^2^, Deborah Robson3, Peter Hajek^4^, Steve Parrott^5^, Catherine Best^2^, Allan Tyler^1^ and Lynne Dawkins^1^

^*^Corresponding author: Sharon Cox. Coxs15@lsbu.ac.uk

Linda Bauld, [linda.bauld@stir.ac.uk](mailto:linda.bauld@stir.ac.uk)

Allison Ford, [a.j.ford@stir.ac.uk](mailto:a.j.ford@stir.ac.uk)

Deborah Robson, [Deborah.j.robson@kcl.ac.uk](mailto:Deborah.j.robson@kcl.ac.uk)

Peter Hajek, [p.hajek@qmul.ac.uk](mailto:p.hajek@qmul.ac.uk)

Steve Parrott, [steve.parrott@york.ac.uk](mailto:steve.parrott@york.ac.uk)

Catherine Best, [Catherine.best2@stir.ac.uk](mailto:Catherine.best2@stir.ac.uk)

Allan Tyler, [tylera@lsbu.ac.uk](mailto:tylera@lsbu.ac.uk)

Lynne Dawkins, dawkinl3@lsbu.ac.uk

**Abstract**

**Background:** A key UK public health priority is to reduce inequalities caused by smoking tobacco. Promoting smoking cessation amongst deprived groups is central to this objective. Smoking rates are exceptionally high amongst the homeless and the harms of smoking are likely to be exacerbated in this group due to poor cardiac and respiratory health and higher incidence of chronic disease. Electronic cigarettes (EC) are the UK’s most popular quit method but their efficacy and effectiveness has not been tested in homeless adults. This study aims to evaluate the feasibility of supplying free e-cigarette starter kits for smoking cessation to smokers accessing homeless centres.

**Methods/Design:** Prospective cohort four-centre feasibility cluster randomised controlled trial. *Participants:* 120 homeless self-reported daily smokers from four centres (N=30 per centre) across the UK. *Intervention:* Usual care (UC) arm will receive advice to quit and sign-posting to the local Stop Smoking Service. The EC arm will receive a starter kit, explanation on use and a fact sheet and can access technical support from trained centre staff. Follow up assessments for both arms will be conducted at 4, 12 and 24 weeks. Qualitative interviews with 24 participants (12 in each arm) including completers and non-completers, and 12 service centre staff (6 in each arm) will be conducted between weeks 4 and 8. *Primary outcomes:* uptake and use of EC provided to smokers accessing homeless centres. Secondary outcomes include participant retention, facilitators and barriers to engagement, service providers’ capacity to support the study, quit rates/cigarette consumption at each follow up time point, participant utilisation of health care services and resources used.

**Conclusion:** If successful, the results will be used to inform the design of a fully powered randomised controlled trial to evaluate the effectiveness of providing free e-cigarette starter kits for smoking cessation to homeless smokers. If the main trial is effective, the service delivery model has the potential to be rolled out nationally across homeless centres. **Trial registration**: Research Registry: researchregistry4346; registration date: 21/08/2018.

**Keywords: homeless; homelessness; smoking cessation; e-cigarettes; inequalities; feasibility, uptake, retention**

**Background**

Tobacco smoking remains a primary cause of preventable illness in the UK, leading to chronic disease, disability and causing an estimated 96,000 deaths a year. Smoking rates in the UK general population continue to decline, reaching an historic low of 15.8% in 2016^1^. However, this masks significant inequalities, with the most deprived groups in society continuing to smoke at high rates with little indication of future decline ^2^. Smoking is a leading cause of health inequality and smoking-related deaths are two to three times higher among disadvantaged groups ^2^. Those in the most deprived groups are more highly nicotine dependent ^3^, make fewer quit attempts and are less successful when they do try to quit ^4,5^. Smoking rates are exceptionally high amongst the homeless in particular (78%)^6^ and the harms of smoking are likely to be exacerbated in this group due to poor cardiac and respiratory health ^7^, higher incidence of chronic disease, risky smoking practices, i.e., puffing harder and longer, smoking unfiltered cigarettes, smoking discarded butts and sharing cigarettes ^8-9^, as well as reporting high levels of illicit substance dependence ^10-11^. In addition to the health impact, homeless smokers may maintain or exacerbate their poor financial situation by continuing to smoke, spending an estimated 20% of their income on cigarettes ^12^.

Engagement with health services including stop smoking services is poor among the homeless ^6,13^, although desire to stop smoking is no different to the general population ^12^. However, there is a paucity of research on smoking and quitting behaviour amongst the homeless in the UK; to date the evidence derives almost entirely from the USA and Australia. Such studies have addressed the provision of smoking cessation support by measuring the benefits of increased access to services on smoking reduction, service up-take, and treatment efficacy ^10,12,14-16^. These have included a range of interventions including: motivational interviewing, cognitive behavioural therapy, nicotine replacement therapy (NRT) and/or other pharmacotherapies ^17-20^. Twenty-six week quit rates are rather low, ranging from 0% - 17%. To date, positive effects of smoking cessation is evidenced with use of NRT and behavioural support only; to our knowledge, there is no evidence on the use and effectiveness of EC in this population although homeless smokers report curiosity about EC ^21^.

Several qualitative studies have provided further insight into the reduced cessation success of smokers in the most deprived groups. A study of 24 low SES smokers in Australia ^22^ suggested that feelings of guilt, shame and stigmatisation contributed to reduced quitting success and acted as an impediment to accessing cessation support. Participants had previously attempted to quit smoking and reported undesirable or unhelpful experiences with treatment services. In addition, a study of 25 homeless smokers’ perspectives on smoking cessation treatments in the US ^23^ reported that they were uninterested in established cessation approaches such as NRT which they viewed negatively and preferred to engage in their own self-defined, alternative smoking interventions, including e-cigarettes (EC).

In the UK, EC are currently the most popular method for quitting^24^ with promising evidence of efficacy for smoking cessation^25^ and health improvements are evident where their use has completely replaced smoking ^26-27^. Those who opt for EC however, tend to be better educated and higher earners ^28^. While in the long-term EC are far cheaper than smoking, they carry an initial start-up cost which may deter those on lower incomes ^29^.

A key public health priority ^30-31^ is to reduce inequalities in health caused by smoking tobacco. Promoting smoking cessation in marginalised and vulnerable groups is central to this objective. In the recent Tobacco Control Plan for England (July 2017)^30^, the Department of Health and Social Care expressed its commitment to evidence-based innovations to support cessation and will seek to support smokers adopting the use of less harmful nicotine products such as EC. Similarly, in the National Institute for Health and Care Excellence (NICE) draft consultation on stop smoking interventions and services guidelines issued last year (September 2017)^32^, a key recommendation for research is the effectiveness and cost-effectiveness of EC in helping people to stop smoking, particularly in disadvantaged groups. To date there have been no published smoking cessation studies focused on the homeless in the UK. Given their popularity for quitting ^24^ and promising evidence of efficacy for smoking cessation ^25^, EC may provide an additional smoking cessation strategy. EC may hold additional value for homeless smokers who tend to show negative attitudes towards, and poor engagement with, traditional Stop Smoking Service (SSS).

This study aims to address a gap in research on the uptake and use of EC for smoking cessation amongst homeless smokers in the UK.

**Objectives**

The overall purpose of the research is to undertake a cluster randomised controlled trial to evaluate the feasibility of supplying free EC starter kits for smoking cessation to smokers accessing homeless centres. Table 1 presents the study objectives and associated outcome measures.

**Table 1: Feasibility study objectives and outcome measures**

| **Objective** | **Outcome measure** |
| --- | --- |
| O1. Assess willingness of smokers to participate in the feasibility study to estimate recruitment rates and inform a future trial. | In both arms, we will record the number of smokers asked to take part and the number who consent. |
| O2. Assess participant retention in the intervention and control groups. | Record a) how many participants are still using e-cigarettes in the intervention group, and b) how many participants complete assessment measures in each arm at each time point. |
| O3. Examine the perceived value of the intervention, facilitators and barriers to engagement and influence of local context. | Qualitative interviews with 4-week completers and non-completers, quitters and smokers (N=24, approx. 6 per site) between weeks 4 and 8 across both arms. |
| O4. Assess service providers’ capacity to support the study and the type of information and training required. | Qualitative interviews with keyworkers and front-line staff (N=12; approx. 3 per site) across both arms. |
| O5. Assess the potential efficacy of supplying free e-cigarette starter kits | Measure breath CO levels, self-reported quit rates/cigarette consumption and HRQoL (using the EQ-5D-3L) at each follow up time point. |
| O6. Explore the feasibility of collecting data on contacts with health care services within this population as an input to an economic evaluation in a full RCT. | Record participant utilisation of primary and secondary health care services using a self-report service-use questionnaire at each time point. |
| O7. Estimate the cost of providing the intervention and usual care. | Record all resources used in the delivery including staff costs, e-cigarettes and other costs incurred. Staff will complete a pro forma to record contact time, non-contact time and other resources used in delivery. |

HRQoL: Health-related quality of life. Carbon Monoxide: CO.

**Study design**

A four-centre feasibility cluster randomised controlled trial with a nested process evaluation qualitative component.

**Methods**

**Participants and recruitment**

An estimated 70-90% of all the attendees who access the participating homeless centres are thought to be smokers. Participants (N=120) will be recruited from four homeless centres (serving as clusters; N=30 participants from each) randomly allocated to EC or usual care (UC) arm. Clients who smoke and are already actively engaging with the service (e.g. in regular attendance, showing signs of making a changes, acting as volunteers etc., as assessed by service centre staff) will be invited for participation. Those agreeing to take part will then be invited to consent and complete a baseline assessment with a member of the research team at their next visit. Although centres will be randomised, participants will receive the same study information sheet and consent form and will complete baseline assessments *before* being told of their randomisation condition.

Those agreeing to take part will then be invited to consent and complete a baseline assessment with a member of the research team at their *next* visit to the homeless centre –no EC will be given out at the first appointment. All assessments will be conducted by the research team and will take place in a quiet room at the centres.

*Inclusion criteria*: aged 18 and over, self-reported daily smoking (confirmed by homeless centre staff and then biochemically verified by exhaled CO at recruitment), currently accessing homeless centre services and actively engaging with the service (determined by homeless centre staff). In order to represent this population of smokers as accurately as possible, we will not exclude participants on the basis of physical/mental health diagnoses or other addictions.

*Exclusion criteria:* non-smokers, currently using another smoking cessation aid, under 18 years, pregnant, unable to consent (e.g. currently intoxicated or unable to speak English); not known to centre staff. We will invite everyone who meets our inclusion criteria at each centre to participate until we have recruited N = 30 at each centre.

**Interventions**

Figure 1 provides a summary of the intervention and data collection points.


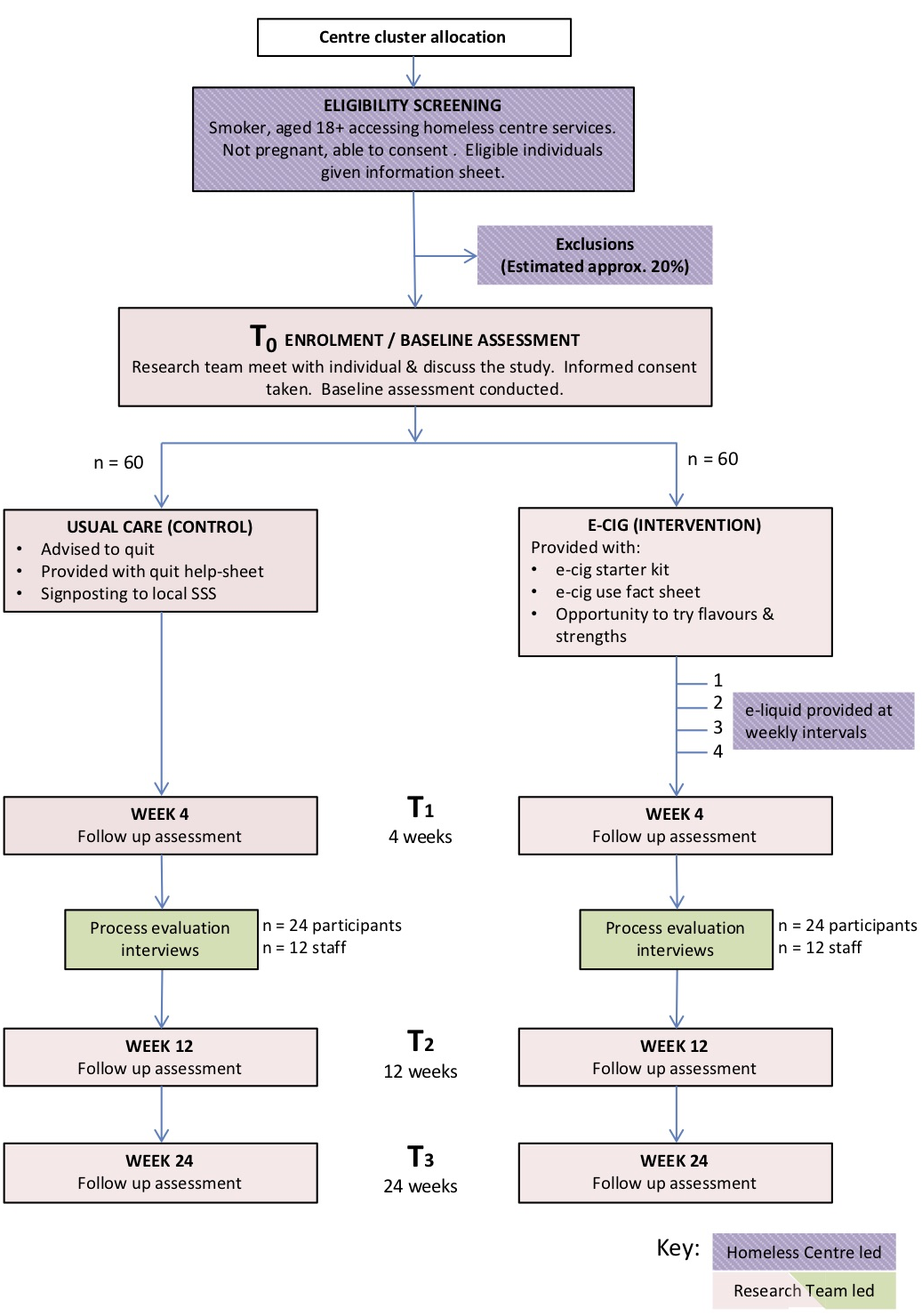


**Intervention Group – EC starter kit**

The intervention has been used in a number of positive trials with smokers in the general population and those with a mental health diagnosis^33-35^. These studies provide proof of concept. However, the proposed trial will be the first in the world (to our knowledge & in consultation with our international colleagues) to explore the feasibility of supplying free EC starter kits to homeless smokers.

Participants in the EC arm will be provided with a starter kit comprising a tank-style refillable EC with a choice of: a) nicotine strength e-liquid (2 options) and b) flavours (3-4 options). They will receive an explanation of how to use the product and a ‘guide to e-cigarettes’ fact-sheet. E-liquids will be supplied for four weeks at weekly intervals by centre staff. Evidence suggests that smokers successfully transition to vaping with higher nicotine e-liquids^36^, thus we have opted for the 2 higher strength e-liquids (12 & 18mg/mL). Evidence also suggests that smokers have different preferences regarding flavours^37^, so a choice of flavours will be offered (e.g. tobacco, menthol, fruit & bakery). Participants will be given time to try different flavours and nicotine strengths at baseline and be permitted to switch between flavours in accordance with documented vaping practices^38^. Homeless centre staff will provide participants with five 10ml bottles of e-liquids per week (approx. 7mL a day) in accordance with the upper level reported in the recent UK national survey ^39^.

To support engagement, participants will be compensated with £15 vouchers for each follow-up assessment and interview as this has been shown to improve retention in other studies with homeless smokers ^40-41^.

*Training & Delivery of the Intervention:*

We will provide training for staff at each centre at the beginning of the recruitment period and ongoing technical support (as required) from the research team.

Consistent with the mode of delivery should the intervention prove efficacious and be adopted into homeless centre practice, the intervention will be delivered by the keyworkers from the homeless centres. Staff will offer EC starter packages to those who provide consent to the Research Assistant. This will be followed by weekly supplies of e-liquid (for 4 weeks) and technical support (assistance with charging, re-filling and replacing atomisers and lost charging leads). Participants will be able to charge their EC at the homeless centres.

Staff will be trained by the research team. Our discussions with staff to date have highlighted the need for training in technical matters relating to EC use (e.g. refilling the tank, replacing atomisers and charging). Prior to training we will ask staff members from all centres to complete a questionnaire on attitudes towards, and knowledge of, EC to inform our training for the feasibility study and the main trial. We will seek to allay any staff concerns about EC use and convey clear evidenced based information about what is known about EC. We will train staff on how to deliver correct advice about EC to participants and will provide a practical hands-on demonstration relating to aspects of use, charging and battery safety. Additional technical support for staff and participants will be available via telephone and an online guide to EC use posted on homeless centre websites and available as posters/flyers at homeless centres.

**Control Group – Usual Care**

Those in the control group (UC; N=60 across two centres) will be recruited in the same way as intervention group (EC) participants and will receive the same study information sheet and consent form. After meeting the researcher to provide baseline information and being informed they are in the UC group, participants will be referred to the keyworker to receive brief advice to quit, and a ‘help-quit’ leaflet including information about the location and opening hours of the local SSS. Paper copies of the help-quit leaflet (with SSS contact details) will be available as posters/flyers at homeless centres.

Control participants will be seen at baseline and followed up at the same time points (4, 12 and 24 weeks) using the same measures as the intervention group. We will monitor uptake/recruitment rate to the control group and the percentage available for follow up. All participants (intervention and control) will be offered a £15 voucher for each follow up and a further £15 voucher for interviews.

**Primary and secondary outcome measures**

Objectives and associated outcome measures are presented in Table 1.

Assessment of the proposed primary and secondary outcomes for the main trial (CO validated, HRQoL) will be conducted at baseline and all follow up time (including quit rates, smoking reduction) points to assess preliminary efficacy of the intervention for smoking cessation. Early (4 week) assessment is essential as most relapse to smoking occurs early in a quit attempt ^1^. Assessment at this point will allow us to address how many participants continue to use EC during and after the free supply period.

**Participant timeline**

This is an 18-month study commencing October 2018. Participant interventions and data timelines are illustrated in Figure 1.

**Sample size**

As this is a feasibility study, a formal power calculation based on detecting evidence for efficacy has not been conducted. However, based on the preliminary scoping work, each homeless centre has contact with between 25 and 120 homeless clients every day, of which 70-90% are likely to be smokers. Other studies in homeless populations have reported follow-up rates ranging between 24% and 88% (depending on the location of visits, provision of incentives & use of prompts, see Richards et al. 2015 ^41^). Therefore, estimating that 50% of those who agree will drop out in the period between consenting to participate and the final follow up at 24 weeks, the sample size at the final follow up could be estimated at 60 minimum. This is a pragmatically chosen sample size, based on the information currently available, to allow us to identify evidence of feasibility, recruitment rates and any problems with the intervention or research methods. An aim of the feasibility study is to calculate the required sample size (and an intra class correlation coefficient (ICC) for a possible future definitive cluster RCT.

A sub-group of 24 homeless smokers (approx. 6 per centre) and 12 staff members (approx. 3 from each centre) will take part in the qualitative process evaluation interviews. Within this sample we will aim to include continuing participants and those who have withdrawn from the study, as well as those in the e-cigarette and usual arms. This sample size is adequate for collection of qualitative data necessary to assess objectives 3 and 4.

**Data collection**

At the first meeting, once the participant has given full written informed consent we will conduct baseline assessments: These will include:

- Demographic information and homeless status/history.
- Smoking status and severity of tobacco dependence, measured by the Fagerstrom Test of Cigarette Dependence (Fagerstrom, 2012) ^42^ and, carbon monoxide (CO) expired breath.
- Smoking history (e.g. length of smoking, previous number of quit attempts, support used; EC past and current use).
- Motivation to stop smoking, measured by the Motivation to Stop Scale^43^, a 7- level single-item instrument which incorporates intention, desire and belief to quit smoking.
- Mental health status, measured using the 9-item Patient Health Questionnaire (PHQ9) ^44^ and the 7-item Generalised Anxiety Disorder (GAD7) questionnaire ^45^.
- Alcohol use, measured using the Alcohol Use Disorders Identification Test (AUDIT) ^46^, a 10-item screening instrument developed by a WHO to screen for a range of drinking problems.
- Drug use measured using The Severity of Dependence Scale (SDS) ^47^, a brief, five-item screening measure of psychological aspects of dependence.
- Health Related Quality of Life (HRQoL) ^48^ measured using the EQ-5D-3L, a widely used measure which provides a single value for health status that can be used in the clinical and economic evaluation of an intervention.

All questionnaires and measures have good psychometric properties and have been used in previous research with vulnerable populations^49-51^. A researcher will be present throughout the session to guide participants in their completion of the measures.

Following the completion of these baseline measures, participants will be told which condition they have been allocated to and then provided with the EC starter kit or UC (according to homeless centre allocation; see section 7 above for details). A follow up appointment will be made, and where participants have consented to providing contact telephone numbers, these will be noted for the purpose of sending text message reminders.

Follow ups at weeks 4, 12 and 24 will collect information on: self-reported smoking abstinence, number of cigarettes smoked, breath CO levels, engagement with the local SSS, use of EC and other tobacco/nicotine containing products, HRQoL and mental health status, adverse effects of /EC use, use of other drugs (including use of the EC for vaping other substances) and use of primary and secondary health care services. We will assess adverse events using a Visual Analogue Scales (VAS) as used in our previous studies. Anyone reporting adverse effects associated with use with be encouraged to discontinue usage. Furthermore, with the participant’s consent, at baseline, we will record the contact details of people involved in their care (e.g. GP, Community Mental Health Team); these will then will be alerted if their PHQ9 scores are initially of concern or increase between assessments. There is standard cut off severity scores 0 – 4 = None/ 5 – 9 =Mild/ 10 – 14= Moderate/ 15 – 19=Moderately Severe/ 20 – 27 =Severe^52^.The client’s keyworker/s will be informed in the event of any negative impact on mental health. Wellbeing of the participant will be monitored by their keyworker and correct protocols from the service centres followed. To further monitor risk and adverse effects, we utilise a purposefully developed unintended consequences checklist (Robson et al., in prep).

**Process evaluation**

Twenty-four participants will be interviewed after the intervention phase of the study (between weeks 4 and 8). Interviews will explore participants’ views on the delivery and perceived value of the intervention, barriers and facilitators to engagement with the study and EC use, and any unintended consequences of participation and supply of the EC (e.g. use of the EC for vaping other substances). Interviews will be conducted face-to-face by a member of the research team, using a semi-structured topic guide. Interviews will last around 40 minutes and will take place in a quiet private space in each of the centres. With participants’ consent, interviews will be digitally recorded and transcribed in full for thematic analysis.

All participants will be asked at enrolment if they are willing to be contacted about taking part in an interview about their experiences of the study. Those that have consented to be contacted and who have been selected for interview based on purposive sampling (see below) will be contacted by a member of the research team, either by telephone or in person at the homeless centre, and invited to take part. Participants will be provided with a participant information sheet and written consent will be obtained. A £15 Love to Shop voucher will be offered in recognition of their contribution to this part of the study.

The views of homeless centre staff (keyworkers & front-line workers who directly support homeless clients and who may also be providing support for the study) will be explored. Semi-structured staff interviews will be conducted face-to-face to investigate how the study has been delivered, perceived barriers and facilitators to engaging with the study and EC use, and any unintended consequences. This will enable us to identify how study processes may be modified or developed for a future trial, including any additional staff support or training required. Up to 3 members of staff in each homeless centre (N=12) will be recruited. Information sheets will be provided and consent obtained.

**Planned Analysis**

For both groups, the number/percentage meeting eligibility criteria, invited to participate, screened and agreeing to participate will be recorded and the proportion of those who meet eligibility criteria who are invited, screened and who agree will be documented. The number/percentage completing the baseline assessment, receiving the intervention or UC, attending each follow up, still engaging with the treatment/UC, and providing outcome data will also be descriptively summarised. The number of participants withdrawing, along with reasons for withdrawal (where possible) will be summarised by group. A CONSORT diagram to illustrate the flow of participant progress through the phases of the EC and UC group will be presented in the final report.

Baseline data (housing situation, age, gender, socioeconomic status , ethnicity, physical and mental health, other drug use, health-related quality of life, motivation to quit, nicotine dependence) will be summarised using frequencies and descriptive statistics and the groups (EC v UC) will be compared using t-test, Chi-squared or Mann Whitney U tests as appropriate.

To assess the potential efficacy of supplying free EC starter kits and to inform the sample size for a future larger trial, we will summarise the number of participants reporting sustained smoking abstinence (CO verified) and a 50% reduction in smoking in each group and at each follow up time point. These variables will be analysed using intention to treat analysis; that is, all those randomised will be included in the analysis as belonging to the group to which they were randomised and those with missing outcome data will be treated as relapsers. The analysis will be by generalised linear mixed effects models as this method will account for the clustering of observations within centres. From these models we will estimate the effect size of the intervention after adjustment for covariates. The ICC will be estimated from the random intercept model. The initial models will also indicate which individual level predictors are important influences on cessation in this population and estimate of their effect size. All the above parameter estimates will be used to calculate the sample size for the full trial. This will be done by simulation in Stata^53^ as this allows complex hierarchical nature of the data to be modelled including covariates. As noted above, our sample size estimation for the main trial at this stage is based on ICCs from the Prescription for Health program^54^.

We will use the results of EQ-5D-3L to calculate QALYs in each group using the area under the curve plotted from baseline and follow up points. We will also present the costs of the programme and analyse the service use questionnaires for completeness. Results from the health economics component will be used to refine the instruments for a full RCT.

The objectives relate to factors considered essential to inform the conduct of a full trial. We have used Thanbane et al’s (2010)^55^ guidelines to assess success and inform our progression criteria. We will continue without modifications to a full trial if the following success criteria are met:

1. At least 50% of eligible participants can be recruited within a 4 week period.
2. No more than 5% of all recruited participants cross from one arm to the other.
3. At least 50% of all recruited participants complete follow up assessments

If the retention rate (those available for follow-up) is between 20-50%, we will assess reasons for this via process evaluation interviews and determine if and how retention and engagement could be improved in a main trial. In this situation, the main trial may be feasible with modifications and/or close monitoring. If less than 20% of participants complete follow up assessments, we will consider the main trail unfeasible and will not proceed with a future larger trial application.

The qualitative data collected from participant and staff interviews will be analysed using a thematic analysis approach^56^. The analysis will be both deductive, identifying themes specified in the semi-structured interview topic guides, and inductive, identifying themes from individuals’ accounts that have not been previously considered. Verbatim transcripts of the interviews will initially be read several times to ensure familiarity with the data and identify emerging themes. A coding framework will then be developed and all transcripts will be systematically coded into themes using an iterative approach. Coded themes will be interpreted and discussed among the research team. Themes will also be compared and contrasted between groups such as completers and non-completers, and participants at different centres to explore any impacts specific to the local context. Coding and analysis will be aided by the qualitative analysis software NVivo 10.

**Patient and public involvement**

We have engaged with multiple agencies as well as service users; from the conception phase through to the study design and management. These processes have involved staff and service users from 7 independent homeless centres.

As part of our scoping work we have collected data from 283 service users accessing homeless support services across England and Scotland, collecting information on current smoking patterns, willingness to be involved in research, randomisation. We have also provided 3 different e-cigarettes to a small number of current smokers accessing one homeless centre in London to gain feedback on which products is most appropriate for the feasibility study. This work has been essential in selecting the most appropriate product.

Furthermore, we have conducted in depth discussions with staff members at the services directly involved in this study regarding the study design. We have chosen a cluster randomised design rather than individual randomisation due to staff concerns regarding cross-contamination and attrition in the control arm if one service user received an e-cigarette and others did not. We have facilitated a discussion group with service centre staff about training requirements to support the e-cigarette arm. Discussion related to data led to several design and procedural changes to our initial ideas. For example, only 30% of homeless smokers surveyed said they would be willing to use NRT. We have therefore opted for a ‘usual care’ (no NRT) control arm.

Lastly we have discussed the study with public health and leading academics in the tobacco field who have agreed to join our advisory group.

**Ethical considerations**

Ethical approval was granted by London South Bank University (LSBU, REF:1821) and has been funded by the National Institute of Health Research (NIHR). The study will comply with the ESRC research ethics framework, the LSBU ethics code of practice and the British Psychological Society (BPS) code of ethics and conduct. We will adhere to the LSBU and the University of Stirling ethical research governance codes and research data management policies. The research will be guided by the four principles set out by LSBU and the BPS: i) Autonomy (every individual has the right to think independently and act freely to decide to participate, continue or withdraw from a research study without hindrance; ii) Beneficence (research must have value to individuals, groups, communities or add to the knowledge base; iii) Non-Maleficence (do no harm); and iv) Justice (research is conducted fairly and with respect for human rights). Action will be taken to uphold all four principles such as clearly and fully informing participants of the likely risks and benefits of participating and obtaining full written consent.

Whilst the potential harm reduction effects of EC are well recognised, the use of EC in this population may pose some risks. In order to systemically monitor risk and adverse effects we will utilise an unintended consequences checklist (designed specifically for the study) that participants and keyworkers will complete at each follow up time point. Staff will also be encouraged to alert the research team if they suspect activity which falls outside of the normal use of the EC to the research team. Regular contact between the research team and the service centre staff will be maintained.

**Discussion**

A key public health priority ^2,30-32^ is to reduce inequalities in health caused by smoking. However, to date there have been no published smoking cessation studies focused on the homeless in the UK and no study worldwide (to date) which has measured the effectiveness of EC versus usual care for smoking cessation in homeless adults. Therefore, this study represents the first to measure the uptake and use of EC provided to smokers accessing homeless centres.

There are a number of benefits in stopping smoking, and the gains of stopping smoking for this population in particular would be significant. These include (but not limited to) improvements in physical and mental health; substantial financial savings; a reduction in communicable disease-risk (by reducing risky smoking^8-9^); and the potential of increasing access to homeless support services which have adopted no-smoking policies.

However, there are notable clinical and environmental factors specific to this population which may challenge the study process. Primary issues relate to engagement and retention. Few studies include homeless smokers in cessation studies but failure to engage with such deprived groups could further exacerbate health inequalities. Retention is also likely to be an issue thus factors likely to promote retention have been introduced - sending text reminders and providing vouchers for follow-up. However, the purpose of the feasibility study is to measure retention to explore these factors in order to determine whether a full trial is likely to be feasible.

Homeless smokers’ circumstances can be unpredictable and are quickly liable to change; support and finances can quickly diminish. In this respect, it is recognised that EC may become lost, damaged, sold or stolen. We will monitor the incidence of such events carefully as part of our feasibility study. There are very few research studies of comparable interventions with comparable populations on which to predict how frequently these unintended consequences might occur. To date there have been two published studies of EC use for smoking cessation in people with severe mental illness^34,57^, and one in methadone users^58^. None of these studies reported that participants lost, sold or had their EC devices stolen. While we will explore and document the reason why devices may have been lost/stolen, we will not provide a replacement. If devices are faulty – due to manufacturing error – these will be replaced.

To add to this, in this population there are also other more superficial concerns such as access to charging. This is likely to differ across the homeless centres involved; whilst clients in residential units will be able to charge their EC in their own room, other centres can only provide charging facilities during the day. An estimated 25-30% of clients attending the study centres are rough sleepers; the majority are in temporary accommodation/sheltered housing or ‘sofa-surfers’ and have access to electricity. EC charging also relies on the availability of a charging plug and lead which are easily lost so extra charging leads will be made available. Such factors will provide richer contextual data relating to the local environmental influences; this will be explored by our qualitative process evaluation interviews.

Overall, we expect very few adverse reactions from EC use. A recent evidence review of e-cigarettes commissioned by Public Health England^59^ included details on adverse reactions of EC from the Medicines and Healthcare products Regulatory Agency (MHRA) Yellow Card Scheme. Very few reports (n=37) about EC were received between 1/1/15 and 20/10/17, compared with 263 reports about suspected adverse drug reaction to licenced nicotine replacement therapy. The most common reported adverse reactions to EC related to gastrointestinal disturbance (e.g. nausea) and respiratory problems (e.g. cough). The PHE evidence review^59^ also reported that research studies that evaluated the effectiveness of EC for smoking cessation, found that smokers who used EC for up to two years did not have an increased health risk compared to smokers who did not use EC.

It is also important to consider co-morbidities in this population in terms of risks. For example, we recognise that our target population will represent a high proportion of adults with a mental health diagnosis. However, based on previous research, stopping smoking has been shown to improve symptoms of mental health^60^; mental health will be monitored using PHQ9 58 and the GAD7 questionnaire.

The role of other substances alongside nicotine and tobacco use, will be monitored; 40% of the homeless misuse other substances^6^ and may be tempted to use the device for other illicit drug. Likewise, the above studies^34,57-58^, did not report that participants used their EC for other purposes than to vape nicotine, e.g. to vape illicit substances. This is a trend that has been described in recent years by drug users in online forums, blogs and videos, though there is very little research on the topic. A recent small sample study in the UK^61^, reported that 39.5% of those using an EC had used it for recreational drug administration in their life time. Most commonly reported was cannabis vaping; 18% of EC users reported lifetime use and 10.6% had used in the last 30 days. Other substances used in EC included (% indicate lifetime use): MDMA/Ecstasy (11.7%), cocaine (10.9%), mephedrone (8.5%), crack (8.4%), synthetic cannabinoids (7.8%) and heroin (7.1%). Regular use of EC for vaping these drugs was not reported. It is possible that use of EC for other drugs may be different in a homeless population, but with a combination of both scarcity of resources and substance dependence it is plausible that illicit drugs would not be used other than the purpose for which they are acquired. Furthermore, this study does not propose to use the types of devices which are amendable to drug vaping, nonetheless this will be carefully monitored.

Another risk is the possible use of incentive vouchers in exchange for other drugs although other research with homeless smokers did not find this to be a significant problem. For example, Baggett et al. (2017)^40^ reported that financial incentives actually increased brief smoking abstinence and quit attempts without worsening substance use.

In order to systemically monitor risk and adverse effects we utilise an unintended consequences checklist that participants and keyworkers will complete at each follow up time point. Staff will also be encouraged to alert the research team if they suspect activity which falls outside of the normal use of the e-cigarette to the research team. Regular contact between the research team and the service centre staff will be maintained.

To summarise, this study will address both the evidence and equality gap on EC use and uptake among the most vulnerable and hard to reach groups in the UK. The main goal of the study is to make a decision as to whether to proceed to a larger trial and how best to run the trial. In this feasibility study we will explore preliminary evidence on whether the provision of free EC starter kits is a) more effective for smoking cessation than usual care and b) cost-effective. The NHS cost of treating smokers is estimated at £2 billion a year in England alone. There is interest in understanding the potential of EC for cost savings via promoting smoking cessation. The intervention may be attractive to service providers if our economic evaluation of a full trial shows the intervention to be cost-effective. If successful, homeless centres may consider adopting this approach to reduce inequalities and the impact of diseases caused by smoking in this vulnerable group. The intervention is highly scalable could be rolled out across homeless centres nationally with no further outlay to the NHS and potential to reduce future NHS costs.

Finally, the authors will work closely with our advisory group to develop a dissemination plan in order to communicate our study findings widely and in an accessible manner to the general public. Data will be presented to the homeless centre staff and clients (including participants in both the e-cigarette and usual care arms) via a verbal presentation and a plain English summary which can be distributed in paper form to clients and via homeless centre webpages. Anonymised data will be available from the funder (NIHR), and by the university repository. We will draw on our existing contacts, social media networks and mailing lists to disseminate findings to other beneficiaries (i.e. SSS, anti-poverty groups, other homeless centres) to maximise the impact of our findings.

**Declarations:**

**Abbreviations**

CO: Carbon Monoxide

EC: Electronic cigarette/s

HRQoL: Health-related quality of life

NIHR: National Institute of Health Research

SSS: Stop Smoking Service/s

UC: Usual care

VAS: Visual analogue scale

ICC: Intra cluster correlation coefficient

**Ethics approval consent to participate**

Ethical approval was granted by London South Bank University (Application reference:1821.) and has been funded by NIHR Public Health (Application reference: 17/44/29).

**Consent for publication**

Not applicable

**Availability of data and material**

Not applicable

**Competing interests**

This project was funded by the NIHR Public Health Research (project ref: 17/44/29). As per the conditions of the grant application review process this study was peer-reviewed by the NIHR.

LB, AF, DR, SP, CB, AT have no competing interests.

SC has provided consultancy work for the Pacific Life insurance group on smoking and reduced risk product use and prevalence rates.

PH received research funding from and provided consultancy to manufacturers of stop-smoking medications.

LD has provided consultancy for the pharmaceutical industry (2015, 2017) and acted as an expert witness for an e-cigarette patent infringement case (2015). Between 2011 and 2013 she conducted research for several independent electronic cigarette companies for which the University of East London received funds. The e-cigarette companies involved had no input into the design, conduct or write up of these projects.

**Funding**

This project was funded by the NIHR Public Health Research (project ref: 17/44/29). As per the conditions of the grant application review process this study was peer-reviewed by the NIHR. The funder did not play a role in the study design nor contribution to this manuscript.

**Authors’ contributions**

LD is the lead investigator and the grant holder. LD and SC conceived the original idea for the project. LD, SC and AF collected the pilot study data. LD, SC, LB, PH, and DR designed the study, refined the methodology and all authors contributed to the grant application. SC is responsible for the day-to-day running and coordination of the project. AF is responsible for the qualitative component; SP for the economic evaluation; CB will be responsible for the data analysis. AT is the researcher for the project (London and Northampton) and responsible for data collection in these areas. LD, SC, DR, AT and AF are responsible for staff training. All authors contributed to and edited earlier drafts of this manuscript. All authors have read and approved the final manuscript.

**Acknowledgements**

We would like to acknowledge staff and service users from the included homeless support centres who have contributed their time and knowledge in developing this project.

**Current status**

This study is ongoing. The study will commence in October 2018; participant recruitment will commence January 2019.

**Author details**

^1^Centre for Addictive Behaviours Research, London South Bank University, 103 Borough Road, London, SE1 0AA.

^2^Insitute for Social Marketing, Faculty of Health Sciences and Sport, University of Stirling, Stirling, FK9 4LA

^3^National Addiction Centre, Addictions Department & CLAHRC South London, HSPRD Institute of Psychiatry, Psychology & Neuroscience, King's College London, Addiction Sciences Building, 4 Windsor Walk, Denmark Hill, London SE5 8BB

^4^ Academic Psychology & Health and Lifestyle Research Unit, Wolfson Institute of Preventive Medicine, Barts and The London School of Medicine and Dentistry
2 Stayner's Road, London, E1 4AH

^5^ Department of Health Sciences, University of York, Seebohm Rowntree Building
University of York, Heslington, York, YO10 5DD

References:

1. Office for National Statistics. Adult smoking habits in the UK: 2016. Available at: <https://www.ons.gov.uk/peoplepopulationandcommunity/healthandsocialcare/healthandlifeexpectancies/bulletins/adultsmokinghabitsingreatbritain/2016> [Accessed 17 July 2017].
2. Harker K, Cheeseman H. 2016. The Stolen Years: The Mental Health and Smoking Action Report.
3. Foulds J, Gandhi KK, Steinberg MB, Richardson DL, Williams JM, Burke MV, Rhoads GG. Factors associated with quitting smoking at a tobacco dependence treatment clinic. *American journal of health behaviour*. 2006; *30*(4): 400-412.
4. Brose LS, McEwen A. Neighbourhood Deprivation and Outcomes of Stop Smoking Support–An Observational Study. *PloS one*. 2016; *11*(1): p.e0148194.
5. Public Health England 2015. Health matters: smoking and quitting in England. Available at: <https://www.gov.uk/government/publications/health-matters-smoking-and-quitting-in-england/smoking-and-quitting-in-england> [Accessed 03 December 2017].
6. Homeless link Health Needs Audit. Available at: <http://www.homeless.org.uk/facts/homelessness-in-numbers/health-needs-audit-explore-data> [Accessed 03 December 2017]
7. Frankish CJ, Hwang SW, Quantz, D.A.R.R.Y.L. The relationship between homelessness and health: An overview of research in Canada. *Finding home: Policy options for addressing homelessness in Canada.* 2009*:*1-21.
8. Aloot CB, Vredevoe DL, Brecht ML. Evaluation of high-risk smoking practices used by the homeless. *Cancer nursing*. 1993; *16*(2): 123-130.
9. Tucker JS, Shadel WG, Golinelli D, Mullins L, Ewing B. Sniping and other high-risk smoking practices among homeless youth. *Drug and alcohol dependence*. 2015; *154*: 105-110.
10. Baggett TP, Rigotti NA. Cigarette smoking and advice to quit in a national sample of homeless adults. *American journal of preventive medicine*. 2010; *39*(2): 164-172.
11. Cookson C, Strang J, Ratschen E, Sutherland G, Finch E, McNeill A. Smoking and its treatment in addiction services: Clients’ and staff behaviour and attitudes. *BMC health services research*. 2014; *14*(1): 304.
12. Arnsten JH, Reid K, Bierer M, Rigotti N. Smoking behavior and interest in quitting among homeless smokers. *Addictive behaviors*. 2004; *29*(6):1155-1161.
13. Department of Health. *Healthcare for single homeless people.* London: Office of the Chief Analyst, Dept. of health, 2010.
14. Connor SE, Cook RL, Herbert MI, Neal SM, Williams JT. Smoking cessation in a homeless population. *Journal of General Internal Medicine*. 2002; *17*(5): 369-372.
15. Okuyemi KS, Thomas JL, Hall S, Nollen NL, Richter KP, Jeffries SK, Caldwell AR, Ahluwalia JS. Smoking cessation in homeless populations: a pilot clinical trial. *Nicotine & Tobacco Research*. 2006; *8*(5): 689-699.
16. Reitzel LR, Kendzor DE, Nguyen N, Regan SD, Okuyemi KS, Castro Y, et al. Shelter proximity and affect among homeless smokers making a quit attempt. *American journal of health behaviour*. 2014; *38*(2): 161-169.
17. Okuyemi KS, Goldade K, Whembolua GL, Thomas JL, Eischen S, Sewali B, et al. Motivational interviewing to enhance nicotine patch treatment for smoking cessation among homeless smokers: a randomized controlled trial. *Addiction*. 2013; *108*(6): 1136-1144.
18. Businelle MS, Poonawalla IB, Kendzor DE, Rios DM, Cuate EL, Savoy EJ, et al. Smoking policy change at a homeless shelter: attitudes and effects. *Addictive behaviors*. 2015; *40*: 51-56.
19. Segan CJ, Maddox S, Borland R. Homeless clients benefit from smoking cessation treatment delivered by a homeless persons’ program. *Nicotine & Tobacco Research*. 2015; *17*(8): 996-1001.
20. Bonevski B, Baker A, Twyman L, Paul C, Bryant J. Addressing smoking and other health risk behaviours using a novel telephone‐delivered intervention for homeless people: A proof‐of‐concept study. *Drug and alcohol review*. 2012; *31*(5): 709-713.
21. Vijayaraghavan M, Hurst S, Pierce JP. A qualitative examination of smoke-free policies and electronic cigarettes among sheltered homeless adults. *American Journal of Health Promotion.* 2017; May;31(3):243-50.
22. Boland VC, Mattick RP, McRobbie H, Siahpush M, Courtney RJ. “I’m not strong enough; I’m not good enough. I can’t do this, I’m failing”: a qualitative study of low-socioeconomic status smokers’ experiences with accessing cessation support and the role for alternative technology-based support. *International journal for equity in health*. 2017 Dec;16(1):196.
23. Collins SE, Orfaly VE, Wu T, Chang S, Hardy RV, Nash A, et al. Content analysis of homeless smokers’ perspectives on established and alternative smoking interventions. International Journal of Drug Policy. 2018 Jan 1;51:10-7.
24. Latest Statistics - Smoking In England (2017). Available at: <http://www.smokinginengland.info/latest-statistics/> [Accessed 05 December 2017].
25. Hartmann‐Boyce J, McRobbie H, Bullen C, Begh R, Stead LF, Hajek P. Electronic cigarettes for smoking cessation. *The Cochrane Library*. 2016.
26. Shahab L, Goniewicz ML, Blount BC, Brown J, McNeill A, Alwis KU, et al. Nicotine, Carcinogen, and Toxin Exposure in Long-Term E-Cigarette and Nicotine Replacement Therapy UsersA Cross-sectional StudyE-Cigarettes and Toxin Exposure. *Annals of internal medicine*. 2017; *166*(6): 390-400.
27. Polosa R, Morjaria J, Caponnetto P, Caruso M, Strano S, Battaglia E, Russo C. Effect of smoking abstinence and reduction in asthmatic smokers switching to electronic cigarettes: evidence for harm reversal. *International journal of environmental research and public health*. 2014; *11*(5): 4965-4977.
28. Weaver SR, Majeed BA, Pechacek TF, Nyman AL, Gregory KR, Eriksen MP. Use of electronic nicotine delivery systems and other tobacco products among USA adults, 2014: results from a national survey. *International journal of public health*. 2016; *61*(2): 77-188.
29. Thirlway F. Everyday tactics in local moral worlds: E-cigarette practices in a working-class area of the UK. *Social Science & Medicine*. 2016; *170*: 106-113.
30. Department of Health (2017). Towards a smoke-free generation: a tobacco control plan for England. Available at: <https://www.gov.uk/government/publications/towards-a-smoke-free-generation-tobacco-control-plan-for-england> [Accessed 8 December 2017].
31. Department of Health (2011). Healthy Lives, Healthy People: A tobacco control plan for England. Available at: <http://www.ncsct.co.uk/usr/pub/DH-control-plan.pdf> [Accessed 6 December 2017].
32. NICE. *Stop smoking interventions and services*. *Draft* for consultation. 2017. Available at: <https://www.nice.org.uk/guidance/gid-phg94/documents/draft-guideline-2> [Accessed 8 Dec 2017].
33. Bullen C, Howe C, Laugesen M, McRobbie H, Parag V, Williman J. et al. Electronic cigarettes for smoking cessation: a randomised controlled trial. *The Lancet*. 2013; *382*(9905): 1629-1637.
34. Caponnetto P, Auditore R, Russo C, Cappello GC, Polosa R. Impact of an electronic cigarette on smoking reduction and cessation in schizophrenic smokers: a prospective 12-month pilot study. *International journal of environmental research and public health*. 2013; *10*(2): 446-461.
35. Caponnetto P, Campagna D, Cibella F, Morjaria JB, Caruso M, Russo C, et al. Efficiency and Safety of an electronic cigarette (ECLAT) as tobacco cigarettes substitute: a prospective 12-month randomized control design study. *PloS one*. 2013; *8*(6):.e66317.
36. Farsalinos KE, Romagna G, Tsiapras D, Kyrzopoulos S, Voudris V. Evaluating nicotine levels selection and patterns of electronic cigarette use in a group of “vapers” who had achieved complete substitution of smoking. *Substance abuse: research and treatment*. 2013; *7*:139.
37. Dawkins L, Turner J, Roberts A, Soar K. ‘Vaping’profiles and preferences: an online survey of electronic cigarette users. *Addiction*. 2013; *108*(6): 1115-1125.
38. Farsalinos KE, Romagna G, Tsiapras D, Kyrzopoulos S, Spyrou A, Voudris V. Impact of flavour variability on electronic cigarette use experience: an internet survey. *International journal of environmental research and public health*. 2013; *10*(12): 7272-7282.
39. ASH (2017). ASH Factsheet: Use of e-cigarettes among adults in Great Britain. Available at: <http://ash.org.uk/information-and-resources/fact-sheets/use-of-e-cigarettes-among-adults-in-great-britain-2017/> [Accessed 6 December 2017].
40. Baggett TP, Chang Y, Yaqubi A, McGlave C, Higgins ST, Rigotti NA. Financial incentives for smoking abstinence in homeless smokers: A pilot randomized controlled trial. *Nicotine & Tobacco Research.* 2017. Aug 18.
41. Richards CM, Sharif F, Eischen S, Thomas J, Wang Q, Guo H, et al. Retention of homeless smokers in the Power to Quit study. *Nic Tob Res.* 2015; *17(9)*: 1104-1111.
42. Fagerstrom K. "Determinants of tobacco use and renaming the FTND to the Fagerstrom Test for Cigarette Dependence." *Nic Tob Res*. 2012; 14(1): 75-78.
43. Kotz D, Brown J, West R. Predictive validity of the Motivation To Stop Scale (MTSS) A single-item measure of motivation to stop smoking. *Drug and Alcohol Dependence*. 2013; 128 (1-2): 15-19
44. Kroenke K, Spitzer RL. The PHQ-9: a new depressions diagnostic and severity measure. *Psychiatric annals.* 2002; 1;32(9):509-15.
45. Sptizer RL, Kroenke K, Williams JB, Löwe B. A brief measure for assessing generalized anxiety disorder: the GAD-7. *Pain Practice.* 2006; 10(4): 294-300.
46. Saunders JB, Aasland OG, Babor TF, Fuente JR, Grant, M. Development of the Alcohol Use Disorders Identification Test (AUDIT): WHO collaborative project on early detection of persons with harmful alcohol consumption - II. *Addiction*. 1993; 88: 791-804
47. Gossop M, Darke S, Griffiths P, Hando J, Powis B, Hall W, et al. The severity of dependence scale (SDS): Psychometric properties of the SDS in English and Australian samples of heroin, cocaine and amphetamine users. *Addiction.* 1995; 90: 607-614
48. The EuroQoL Group. EuroQoL-a new facility for the measurement of health related quality of life. *Health Policy.* 1990;16(3):199-208.
49. Torchalla I, Strehlau V, Okoli CT, Li K, Schuetz C, Krausz M. Smoking and predictors of nicotine dependence in a homeless population. *Nicotine & Tobacco Research.* 2011;13(10):934-942.
50. Freeman TP, Stone JM, Orgaz B, Noronha LA, Minchin SL, Curran HV. Tobacco smoking in schizophrenia: investigating the role of incentive salience. *Psychological Medicine.* 2014;44(10):2189-2197.
51. Keogh C, O’Brien KK, Hoban A, O’Carroll A, Fahey T. Health and use of health services of people who are homeless and at risk of homelessness who receive free primary health care in Dublin. *BMC Health Service Review.* 2015;15: 58.
52. The IAPT Data Handbook (2011) Guidance on recording and monitoring outcomes to support local evidence-based practice. IAPT Crown Copyright
53. Feiveson A. Power by simulation. *Stata J.* 2002; 2**:** 107–124
54. Thompson D, Fernald DH, Mold JW. Intraclass correlation coefficients typical of cluster-randomized studies: Estimates from the Robert Wood Johnson Prescription for Health Projects. Annals of Family Medicine. 2012: 10(3): 235-240.
55. Thabane L, Ma J, Chu R, Cheng J, Ismaila A, Rios LP, et al. A tutorial on pilot studies: the what, why and how. *BMC medical research methodology.* 2010; *10(1):* 1.
56. Braun V, Clarke V. *Using thematic analysis in psychology*. Qualitative Research in Psychology. 2006; 3 (2): 77-101. ISSN 1478-0887.
57. Pratt SI, Sargent J, Daniels L, Santos MM, Brunette M. Appeal of electronic cigarettes in smokers with serious mental illness. Addict Behav. 2016;59:30-4.
58. Stein MD, Caviness C, Grimone K, Audet D, Anderson BJ, Bailey GL. An open trial of electronic cigarettes for smoking cessation among methadone-maintained smokers. Nicotine Tob Res. 2016;18(5):1157-62.
59. McNeill A, Brose LS, Calder R, Bauld L & Robson D. *Evidence review of e-cigarettes and heated tobacco products. A report commissioned by Public Health England*. 2018. London: Public Health England. Crown Copyright
60. Taylor G, McNeill A, Girling A, Farley A, Lindson-Hawley N. Changes in mental health after smoking cessation: systematic review and meta-analysis. *British Medical Journal.* 2014; *348:* 1151-1163.
61. Blundell M, Dargan P, Wood, D. A cloud on the horizon- a survey into the use of electronic vaping devices for recreational drug and new psychoactive substances (NPS) administration. *International Journal Medicine.* 2017; Oct: 1-6.
