# Supplementary material for: A cluster feasibility trial to explore the uptake and use of e-cigarettes versus usual care offered to smokers attending homeless centres in Great Britain
Source: PLoS One. 2020 Oct 23;15(10):e0240968. doi: 10.1371/journal.pone.0240968 (PMC7584191; doi:10.1371/journal.pone.0240968)
Supplement: Attachment — Submitted filename: renamed_af8f6.docx [file pone.0240968.s008.docx]

**COMMENTS – Manuscript PONE-D-20-19557**

Title: “A cluster feasibility trial to explore the uptake and use of e-cigarettes versus usual care offered to smokers attending homeless centres in Great Britain”

**Important note: This review pertains only to ‘statistical aspects’ of the study and so** **‘****clinical aspects’ [like medical importance, relevance of the study,** **‘clinical significance and implication(s)’ of the whole study, etc.] are to be evaluated [should be assessed] separately/independently. Further please note that any ‘statistical review’ is generally done under the assumption that (such) study specific methodological [as well as execution] issues are perfectly taken care of by the investigator(s). This review is not an exception to that and so does not cover clinical aspects {however, seldom comments are made only if those issues are intimately / scientifically related & intermingle with ‘statistical aspects’ of the study}. Agreed that ‘statistical methods’ are used as just tools here, however, they are vital part of methodology [and so should be given due importance].**

**COMMENTS:** Since this is a ‘feasibility trial’, sample size is not a big issue anyway. However, the objectives (given later on page-6) are not limited to (feasibility) use of e-cigarettes but comparison with usual care [offered to smokers attending homeless centres in Great Britain] is included. On page 15 [in Sample size section] it is said that “As this is a feasibility study, a formal power calculation based on detecting evidence for efficacy was not conducted”.

Objective three is “3.	Assess the potential efficacy of supplying free e-cigarette starter kits to determine the required sample size for a main trial”. Intra class correlation coefficient (ICC) found in this trial will help to estimate the required sample size for a possible future definitive cluster RCT is understandable [however, need to mention this clearly]. But (because further you also said so) ‘how can the aim of the feasibility study was to calculate the required sample size?’ (please correct the sentence there).

Just a clarification – you said in ‘Methods section of Abstract’ that: “In this feasibility cluster trial, four homeless centres in Great Britain were pragmatically allocated to either a Usual Care (UC) or E-Cigarette (EC) arm” means the allocation was ‘non-random’ (in simple words). Is not that so? [dictionary meaning of ‘pragmatically’ is practically or rationally (therefore, what you said is perfectly alright) but we often describe allocation in terms of ‘random/non-random’]. Therefore, it is desirable to describe/classify allocation procedure in those terms.

Please note [for most data in this article through-out] that though measures/tools used are appropriate, most of them yield data in [at the most] ‘ordinal’ level of measurement [and not in ratio level of measurement as the score two times higher does not indicate presence of that parameter/phenomenon as double (for example, a Visual Analogue Scales {VAS} score or say ‘depression’ score)]. Then application of suitable non-parametric test(s) is/are indicated/advisable [even if distribution may be ‘Gaussian’ (i.e. normal)]. Note also that:

For ‘ordinal’ categorical data [as usual Chi-square test which otherwise is very versatile, the ‘ordering’ in categories is not recognized (example: instead of taking VAS scores are groups as No, Mild, Moderate, Severe, even if you take group sequence as Moderate, No, Mild, Severe, Chi-square value will be same), one variation called ‘Chi-square trend’ test is oftentimes recommended but] most recommended is RIDIT analysis [Bross IDJ. ‘How to use RIDIT analysis’. Biometrics, 1958; 14:18-38. There are many recent references, but they are on application results. This is (an original, old classic and) gives details on ‘how to use’ the technique].

In {last paragraph of} ‘Data analysis’ section, since it is stated that “For assessment of the relationship between other variables and outcomes, cases with missing data were excluded from the analysis“ I would like to know ‘how many cases were available?’. I doubt if ‘what is reported in Table 2’ [table titled as: Unintended consequences of E-cigarette use at 4, 12 and 24 weeks] are ‘consequences of E-cigarette use’. Findings of Table 4 [table titled as: Mental health and substance use at baseline and each follow up time point for the e-cigarette (EC) and usual care (UC) arms] are not properly interpreted, in my opinion {and displaying figures for ‘total’ is relevant, I guess}.

Note that when reported ICC, it is desirable to give the interpretation of ‘intraclass correlation coefficient’s point estimate is 0.0157?’ [though this Fleiss-Cuzick method is little known]. Nevertheless, there are few appreciable points, for example, inclusion of ‘Main RCT sample size calculation’ and discussion on ‘limitations’ [page 35].
