# Supplementary material for: A cluster feasibility trial to explore the uptake and use of e-cigarettes versus usual care offered to smokers attending homeless centres in Great Britain
Source: PLoS One. 2020 Oct 23;15(10):e0240968. doi: 10.1371/journal.pone.0240968 (PMC7584191; doi:10.1371/journal.pone.0240968)
Supplement: Attachment — Submitted filename: responses to reviewers.docx [file pone.0240968.s009.docx]

*Thank you to the reviewers for their kind consideration of our manuscript and suggestions for improvement and clarification. Please find our responses detailed below.*

Reviewer #1: PONE-D-20-19557
Review of the paper A cluster feasibility trial to explore the uptake and use of e-cigarettes versus usual care offered to smokers attending homeless centers in UK

Global assessment
The proposed article is quite long and presents only a prospective limited randomized by cluster feasibility study which does not in itself allow definitive conclusions. On the principle it is the publisher's choice whether or not to accept this type of publication.

But this publication if of interest. This randomized trial concern:
1- tobacco dependence, the leading cause of preventable death. Tobacco related disease and mortality particularly affects vulnerable populations.
2- place of e-cigarette use for tobacco cessation is the object of strong discussions because of the lack of good data’s.
3- an innovative approach in precarious homeless people to quitting smoking through e-cigarettes is welcome
4- numerous very practical point for the conception of a large randomized trial are assessed in this paper.

This important article opens a door to the evaluation of e-cigarettes for nicotine replacement smoked in poor people who are heavy smokers and opens the door to improving health and reducing inequalities. The paper does not provide a definitive result but offers interesting results to launch a large essay that may provide the answer. This article provides an unprecedented series of information to understand the place of e-cigarettes for smokers in great social precariousness.

*Thank you to the reviewer for these positive comments on our manuscript particularly relating to its novelty value and the importance of focusing on this disadvantaged, hard to reach to group. As the reviewer noted, the aim of the study was to determine whether it would be feasible to progress to a main trial (rather than to provide definitive data on efficacy) and we have indeed, learned a lot about working with this group and how to make adaptions for a full trial. As noted below, we have shortened the introduction. The results and discussion have not been shortened as we feel that this is all important information for the reader given the dearth of research in this area.*

In detail

Length of introduction

The introduction (as the paper) is long and could be shortened.

*We have shortened the introduction.*

Randomization page 8
The randomization process and result is not clear and had to be clarified.
It is clear that the randomization concern center and that all smokers in a same center received the same treatment.
• It seem that day centers and 24 h centers are associated in the random process.
• It appear than a center was changed after randomization.
• It appear than the smokers of a center had information of the result of the randomization in the center before inclusion.
• It seem than a center was designed to offer e-cigarette because of the proximity of the study center
I understand that the treatment was allocated by center, but the mode of distribution of treatment by center was not clearly identified (this is not a severe problem for a pilot study and lesson of this trial could be useful for a full size trial), but there is a need to better explain with coherence the allocation of treatment.

*The detail we provided here probably caused more confusion than clarification and was unnecessarily detailed. We have therefore shortened and re-written this section to explain that the allocation was not random (i.e. a deviation from protocol) and was based on centre availability.*

Page 9 recruitment of patients
It would be interesting to clarify what is said to smokers? Is the agreement for a smoking cessation need to be included? Is smoking reduction and option?
Is it clear to the patient that the trial is randomized?

*Any smoker interested in using an EC to try to quit smoking or reduce their smoking was eligible to take part. There was no agreement that a cessation attempt should be made and participants didn’t need to be motivated to quit. Yes, participants were aware that they would be allocated to either an EC or UC group; this was stated on the Participant Information Sheet. This information has been added under ‘Participants and Recruitment’ on page 9.*

Page 11 baseline (and follow-up) measure
The number of questionnaires included in the protocol addressed to homeless subjects is very high. Did the subjects complete the questionnaires on their own, were they completed with the help of a caregiver or by a signatory

*Yes, there were a lot of questionnaires and we did receive feedback (which will be written up as part of our qualitative process evaluation) that this was a burden on participants (this will help to inform the design of our main trial). The researcher read the questions out to participants so they did not complete them on their own and participants were offered several breaks throughout the session. This is explained in our published protocol so we have not repeated this information here.*

NHS Treatement
Please precise what is an NHS treatment (Patch + oral nicotine) varenicline ?

*There is no page number mentioned here to guide us as to where we mention ‘NHS Treatment’ and a search through the manuscript finds no reference to NHS Treatment.*

Page 17 number of subjects in each arm
is there such a difference in the number of subjects in each arm (1/3 more subjects in the e-cigarette group than in the usual treatment group)?

*This was simply due to centre differences. Recruitment was far more successful at the EC day care centre in Northampton. Breakdown of recruitment by centre is shown in supplementary table 1 and this point is noted in the discussion (page 31).*

Table 5 right column 1 to 6
I do not understand that in the right column we have frequencies at zero and percentages which are not zero for the first 6 lines. Please explain more or correct

*Thank you for spotting this! This has been corrected.*

Page 21 retention
I understand than at Week 4 only 25% of patients had reach SSS (no information on treatment) then in the EC arm 75% or 95% of patients use e-cigarette. But there is no or few data of the nicotine received daily in the two groups. Such information on daily dose and duration of nicotine received is important to assess in a complete trial the efficacy of treatment.

*We did collect information about nicotine products (types and weeks) received from the SSS (and other sources) and the number of bottles of e-liquid supplied. This information is presented in supplementary tables 3 and 4 respectively. It was not our intention for this feasibility study to examine daily dose. We will collect detailed information about products used and duration of use if we are funded for a main trial.*

Page 29 cost of intervention
The duration of the visits seems short. The questionnaire completion time is often long in these homeless patients. 5 to 8 minutes for the follow-up visits seems very short, if not impossible, if this time includes the time for completing the questionnaire. If it is the health professional's time indicated: please specify the nature of these times

*Sorry for the confusion. What we referred to was not questionnaire completion time. This is just the keyworker’s time for enquiring participants’ use of EC, giving out e-liquid and providing guidance if needed. Since we only need to cost out the components of the intervention that would be rolled out in practice, research activities such as the researcher’s time/time to complete assessments was not included. The time for visits 1-3 was short because it was mainly just a case of giving out e-liquids. We have clarified this in the second paragraph on page 29.*

The cost needs to obtain one smoking cessation could be pertinent to be calculated in e-cig group. Not in UC group because no a single success.

*Sorry we are slightly confused by the comment. If we understand correctly, the reviewer asked for a cost per quitter in the EC arm. While we acknowledge that cost per quitter would be a pertinent measure for smoking cessation, we felt that to estimate cost per quitter based on such a small sample of a feasibility trial might be misleading, as readers might pick up on the numbers without considering the context. We therefore decided not to report cost per quitter in this study. If we misunderstand this comment, please let us know.*

Reviewer #2: Important note: This review pertains only to ‘statistical aspects’ of the study and so ‘clinical aspects’ [like medical importance, relevance of the study, ‘clinical significance and implication(s)’ of the whole study, etc.] are to be evaluated [should be assessed] separately/independently. Further please note that any ‘statistical review’ is generally done under the assumption that (such) study specific methodological [as well as execution] issues are perfectly taken care of by the investigator(s). This review is not an exception to that and so does not cover clinical aspects {however, seldom comments are made only if those issues are intimately / scientifically related & intermingle with ‘statistical aspects’ of the study}. Agreed that ‘statistical methods’ are used as just tools here, however, they are vital part of methodology [and so should be given due importance].

COMMENTS: Since this is a ‘feasibility trial’, sample size is not a big issue anyway. However, the objectives (given later on page-6) are not limited to (feasibility) use of e-cigarettes but comparison with usual care [offered to smokers attending homeless centres in Great Britain] is included. On page 15 [in Sample size section] it is said that “As this is a feasibility study, a formal power calculation based on detecting evidence for efficacy was not conducted”. Objective three is “3. Assess the potential efficacy of supplying free e-cigarette starter kits to determine the required sample size for a main trial”. Intra class correlation coefficient (ICC) found in this trial will help to estimate the required sample size for a possible future definitive cluster RCT is understandable [however, need to mention this clearly]. But (because further you also said so) ‘how can the aim of the feasibility study was to calculate the required sample size?’ (please correct the sentence there).

*Yes we agree that it requires clarification. We have edited the manuscript to make it clearer that the aim is not to demonstrate the efficacy of the intervention. The aim here is to estimate the parameters necessary to make sample size calculations for a full trial (as suggested by Sim 2019). Of particular importance are the intraclass correlation coefficient and the control group outcome rate. The second paragraph of the abstract has been changed to* ‘The objective of this trial was to assess the feasibility of supplying free e-cigarette starter kits to smokers accessing homeless centres and to estimate parameters to inform a possible future larger trial.’

Sim, J. Should treatment effects be estimated in pilot and feasibility studies?. *Pilot Feasibility Stud* **5,** 107 (2019). <https://doi.org/10.1186/s40814-019-0493-7>

Just a clarification – you said in ‘Methods section of Abstract’ that: “In this feasibility cluster trial, four homeless centres in Great Britain were pragmatically allocated to either a Usual Care (UC) or E-Cigarette (EC) arm” means the allocation was ‘non-random’ (in simple words). Is not that so? [dictionary meaning of ‘pragmatically’ is practically or rationally (therefore, what you said is perfectly alright) but we often describe allocation in terms of ‘random/non-random’]. Therefore, it is desirable to describe/classify allocation procedure in those terms.

*The word ‘pragmatically’ has been changed to ‘non-randomly’ in the abstract.*

Please note [for most data in this article through-out] that though measures/tools used are appropriate, most of them yield data in [at the most] ‘ordinal’ level of measurement [and not in ratio level of measurement as the score two times higher does not indicate presence of that parameter/phenomenon as double (for example, a Visual Analogue Scales {VAS} score or say ‘depression’ score)]. Then application of suitable non-parametric test(s) is/are indicated/advisable [even if distribution may be ‘Gaussian’ (i.e. normal)].

*Thank you for this comment. We have changed the analysis for the continuous variables that can be considered ordinal ie GAD7, PHQ9, to non-parametric Mann Whitney U tests rather than t-tests. We have also changed the measure of dispersion and central tendency for these variables to median and IQR. Corrections to p values have been made in Table 1 but this didn’t change the overall pattern of significant group differences. We note that many studies in psychiatry treat depression and anxiety scales used here (the GAD 7, PHQ9) as continuous ratio scales for example*

*Richards DA, Hill JJ, Gask L, et al. Clinical effectiveness of collaborative care for depression in UK primary care (CADET): cluster randomised controlled trial. BMJ. 2013;347:f4913. Published 2013 Aug 19. doi:10.1136/bmj.f4913*

*Freeman et al The effects of improving sleep on mental health (OASIS): a randomised controlled trial with mediation analysis Lancet Psychiatry. 2017 Oct; 4(10): 749–758.* [*https://www.thelancet.com/journals/lanpsy/article/PIIS2215-0366(17)30328-0/fulltext*](https://www.thelancet.com/journals/lanpsy/article/PIIS2215-0366(17)30328-0/fulltext) *.*

*Finally, we have also taken out the variable relating to amount of cannabis smoked per day as we realised that this was not measured exactly the same way across baseline.*

Note also that:
For ‘ordinal’ categorical data [as usual Chi-square test which otherwise is very versatile, the ‘ordering’ in categories is not recognized (example: instead of taking VAS scores are groups as No, Mild, Moderate, Severe, even if you take group sequence as Moderate, No, Mild, Severe, Chi-square value will be same), one variation called ‘Chi-square trend’ test is oftentimes recommended but] most recommended is RIDIT analysis [Bross IDJ. ‘How to use RIDIT analysis’. Biometrics, 1958; 14:18-38. There are many recent references, but they are on application results. This is (an original, old classic and) gives details on ‘how to use’ the technique].

*The tests presented in Table 1 are intended to show whether allocation at the level of homeless unit resulted in uneven distribution of characteristics between the two groups (e-cigarette versus usual care). Therefore, we do not necessarily wish to test directional hypotheses. For example, in a four category ordinal measure if the control group participants were mainly in the two most extreme categories (highest and lowest) and the intervention groups participants were mainly in the middle two categories this would be of interest even were there no difference in the overall trend ( to being in higher or lower categories overall) between groups. For this reason we have used tests appropriate for categorical outcomes.*

*In some cases we have made inference about the direction of differences for example in the case of the GAD 7 score but this is on the basis of the Mann Whitney U test which does allow directional interpretation.*

*The results for the VAS measures of positive and negative experiences of e-cigarettes, presented in Tables 2 & 3 are descriptive and no inferential statistical analyses have been performed*.

In {last paragraph of} ‘Data analysis’ section, since it is stated that “For assessment of the relationship between other variables and outcomes, cases with missing data were excluded from the analysis“ I would like to know ‘how many cases were available?’.

*This information is included in each of the tables, either in the table header, the table itself or in the table footnote.*

I doubt if ‘what is reported in Table 2’ [table titled as: Unintended consequences of E-cigarette use at 4, 12 and 24 weeks] are ‘consequences of E-cigarette use’.

*We have changed the title of Table 2 to ‘E-cigarette Events’*

Findings of Table 4 [table titled as: Mental health and substance use at baseline and each follow up time point for the e-cigarette (EC) and usual care (UC) arms] are not properly interpreted, in my opinion {and displaying figures for ‘total’ is relevant, I guess}.

*On consideration of this comment we have decided to remove the inferential analysis from table 4.*

Note that when reported ICC, it is desirable to give the interpretation of ‘intraclass correlation coefficient’s point estimate is 0.0157?’ [though this Fleiss-Cuzick method is little known]. Nevertheless, there are few appreciable points, for example, inclusion of ‘Main RCT sample size calculation’ and discussion on ‘limitations’ [page 35].

*We have added a section on the interpretation of the ICC ading on page 27 ‘*The intra class correlation coefficient is the ‘proportion of the total variance in the outcome attributable to variance between clusters’ (Wu, Crespi and Wong 2012). Although the estimated ICC is relatively small (Thompson, Fernald and Mold 2012) neglecting to factor this in to the sample size calculation for a full scale cluster randomised controlled trial would result in reduced statistical power.’

Reviewer #3: This preliminary work provides important indications for a larger study to explore the feasibility of supplying free EC starter kits, targeting a vulnerable population, particularly at risk of smoking but also difficult to study rigorously in a clinical trial.

The questions are relevant and treated from a pragmatic angle.
For example, in practice, certain material challenges will have to be anticipated as best as possible such as, for example, the breakage of E-cigarettes...

The relatively encouraging results give an indication of the strengths and weaknesses of future studies, in particular the high proportion of those lost to follow-up during the study.

More generally, we must welcome the interest of a pragmatic approach that does not make guilt or infantilize towards a precarious and sensitive population.

*We thank reviewer 3 for these encouraging remarks.*
